# Editorial: Pharmacovigilance and drug repositioning research using pharmacoepidemiology

**DOI:** 10.3389/fphar.2023.1225909

**Published:** 2023-06-26

**Authors:** Yoshihiro Noguchi, Miao Yan, Satoshi Yokoyama, Elisabetta Poluzzi

**Affiliations:** ^1^ Laboratory of Clinical Pharmacy, Gifu Pharmaceutical University, Gifu, Japan; ^2^ Department of Pharmacy, The Second Xiangya Hospital of Central South University, Changsha, Hunan, China; ^3^ Toxicology Counseling Center of Hunan Province, Changsha, Hunan, China; ^4^ International Research Center for Precision Medicine, Transformative Technology and Software Services, Changsha, Hunan, China; ^5^ Division of Drug Informatics, School of Pharmacy, Kindai University, Higashi-osaka, Japan; ^6^ Pharmacology Unit, Department of Medical and Surgical Sciences (DIMEC), Alma Mater Studiorum-University of Bologna, Bologna, Italy

**Keywords:** pharmacovigilance, drug repositioning, pharmacoepidemiology, adverse events, drug-drug interactions (DDI), synergy pharmaceutical science

## Introduction

Pharmacoepidemiology, the study of the use and effects of medicines in large human populations, is a bridging science between clinical pharmacology and epidemiology. Pharmacovigilance is “the science and activities related to the detection, evaluation, understanding, and prevention of adverse effects or other problems associated with medicines."

This Research Topic contains six manuscripts using pharmacoepidemiological methods, five original research papers, and one perspective paper, including one paper using electronic health records as an information source, two papers using spontaneous reporting systems, and three papers using nationwide medical information based on claims data.

First, a retrospective cohort study using electronic medical records was reported by (Alsowaida et al.). The global epidemic of COVID-19 has made the development of effective drugs for the treatment and prevention of COVID-19 a global priority. Several post-marketing studies have reported significant bradycardia with remdesivir administration, and this article provides validation in an evidence-based source.

The spontaneous reporting system is one of the most important sources of information in pharmacovigilance, enabling the detection of unknown adverse events, and many safety signals have been reported through the spontaneous reporting system (Fusaroli et al, 2022; Xia et al, 2023). However, many cases registered in the spontaneous reporting system are spontaneously reported and therefore contain various reporting biases (Noguchi et al, 2021). In addition, the use of patient background information is controversial.

Although the risk of adverse events from drug exposure during pregnancy is usually investigated in study designs that include a control group, such as cohort studies, these studies require a sufficient number of cases. They can be time-consuming and labor-intensive to conduct. In some cases, there are few studies establishing short- and long-term safety in pregnancy, including retrospective studies, observational studies and prospective registry analyses (van De Ven et al, 2020). Sakai et al. reported safety signals for drug exposure during pregnancy using a spontaneous reporting database.

Onda et al. used multiple logistic regression analysis to adjust for patient background in the disproportionality analysis. Their logistic regression analysis showed that adding folic acid (FA) to methotrexate (MTX)-based therapy could help reduce the dose-dependent adverse events of MTX, providing clinical evidence to support the beneficial effect of FA.

Taiwan’s National Health Insurance (NHI) program covered more than 99.9% of the 14 Taiwanese population by 2014 (Lin et al, 2018). The NHI Research Database (NHIRD) records residence, sex, age, salary, prescription, medical procedures and disease diagnosis according to the International Classification of Diseases. Lin et al. hypothesize that statins inhibit MSU-induced gout flares through their anti-inflammatory properties, and a cohort study using the 2000 Longitudinal Generation Tracking Database (LGTD 2000), a randomly selected dataset of 2 million NHI recipients, found that statins have chemopreventive potential against MSU.

Yen et al. recruited participants with type 2 diabetes mellitus (T2DM) and cirrhosis from the NHIRD between 1 January 2000 and 31 December 2017 and followed them until 31 December 2018. This report found that using alpha-glucosidase inhibitors was associated with a reduced risk of mortality, hepatocellular carcinoma, compensated cirrhosis, and liver failure in patients with diabetes and compensated cirrhosis.

This type of study has not been conducted exclusively in Taiwan. The Pharmaceuticals and Medical Devices Agency (PMDA), the Japanese regulatory authority, conducts various pharmacoepidemiological studies based on actual data from its medical information database for post-marketing drug safety evaluation. Shida et al. carefully explained the details of these studies.

Since various medical information is a source of pharmacoepidemiological studies, researchers should properly characterize the sources and consider the study design.

The inverse signals detected by pharmacoepidemiological studies are known to be helpful in the search for drug candidates (Kinoshita et al, 2020), and many papers have been submitted on this Research Topic. However, unfortunately, none have been accepted for publication. We hope that research in this area will develop in the future.
